# Electronic Cigarettes and Vaping: A New Challenge in Clinical Medicine and Public Health. A Literature Review

**DOI:** 10.3389/fpubh.2013.00056

**Published:** 2013-11-18

**Authors:** Dominic L. Palazzolo

**Affiliations:** ^1^Department of Physiology and Pharmacology, DeBusk College of Osteopathic Medicine, Lincoln Memorial University, Harrogate, TN, USA

**Keywords:** electronic cigarettes, nicotine addiction, nicotine replacement, smoking cessation, vaping, harm reduction

## Abstract

Electronic cigarette (e-cigarette) use, or vaping, in the United States and worldwide is increasing. Their use is highly controversial from scientific, political, financial, psychological, and sociological ideologies. Given the controversial nature of e-cigarettes and vaping, how should medical care providers advise their patients? To effectively face this new challenge, health care professionals need to become more familiar with the existing literature concerning e-cigarettes and vaping, especially the scientific literature. Thus, the aim of this article is to present a review of the scientific evidence-based primary literature concerning electronic cigarettes and vaping. A search of the most current literature using the pubmed database dating back to 2008, and using electronic cigarette(s) or e-cigarette(s) as key words, yielded a total of 66 highly relevant articles. These articles primarily deal with (1) consumer-based surveys regarding personal views on vaping, (2) chemical analysis of e-cigarette cartridges, solutions, and mist, (3) nicotine content, delivery, and pharmacokinetics, and (4) clinical and physiological studies investigating the effects of acute vaping. When compared to the effects of smoking, the scant available literature suggests that vaping could be a “harm reduction” alternative to smoking and a possible means for smoking cessation, at least to the same degree as other Food and Drug Administration-approved nicotine replacement therapies. However, it is unclear if vaping e-cigarettes will reduce or increase nicotine addiction. It is obvious that more rigorous investigations of the acute and long-term health effects of vaping are required to establish the safety and efficacy of these devices; especially parallel experiments comparing the cardiopulmonary effects of vaping to smoking. Only then will the medical community be able to adequately meet the new challenge e-cigarettes and vaping present to clinical medicine and public health.

## Introduction

Use of electronic cigarettes (e-cigarettes), referred to as vaping, is a relatively new phenomenon that is quickly gaining the interest of many long-time tobacco smokers. According to a report by UBS Securities LLC (1), sales from the e-cigarette market doubled from $250 to $500 million between 2011 and 2012, and are expected to quadruple by 2014. E-cigarettes are becoming a preferred alternative for nicotine delivery among many smokers because of their realistic look, feel, and taste compared to traditional cigarettes. Furthermore, many cigarette smokers have turned to vaping because e-cigarette vendors have previously marketed their product as a cheaper and safer smokeless alternative to traditional cigarettes, and a possible smoking cessation tool. The Food and Drug Administration (FDA) rejected these claims, and in September of 2010 they informed the President of the Electronic Cigarette Association (2) that warning letters had been issued to five distributors of e-cigarettes for “violations of good manufacturing practices, making unsubstantiated drug claims, and using the devices as delivery mechanisms for active pharmaceutical ingredients.” Many web sites still claim that use of e-cigarettes is safe because tobacco is not burned and hence there is no inhalation of the many toxins found in cigarette smoke. For example, Electronic Cigarette Consumer Reviews (3), an e-cigarette website, is filled with anecdotal consumer claims relating how e-cigarettes helped them to quit smoking and improved their overall health.

The FDA has reported that e-cigarette cartridges and solutions contain nitrosamines, diethylene glycol, and other contaminants potentially harmful to humans (4). From their analysis, the FDA reasons that the sale of e-cigarettes should be prohibited or regulated as dangerous nicotine delivery systems that comply with the safety standards of the Federal Food Drug and Cosmetic Act (FDCA) (5). This presents an obvious dilemma since traditional cigarettes, which include nicotine, are proven to be harmful to human health, but are exempt from the FDCA safety standards. After Smoking Everywhere, Inc., filed an injunction against the FDA for restricting the sale of their e-cigarettes in the United States (6), the US Court of Appeals (7) decided that e-cigarettes may not be marketed as a safer alternative to cigarettes, or as a smoking cessation device, but instead must be sold as a smokeless tobacco product subject to the same rules and regulations of other tobacco products. What makes this ruling so controversial is that e-cigarettes contain no tobacco other than a miniscule quantity found in the tobacco flavoring. Despite the court’s decision, e-cigarette vendors have embraced this ruling, and are happy to sell their devices as alternatives to conventional cigarettes, so long as the FDA does not interfere with the sale of their products. Nevertheless, the potential harmful effects of vaping have led the FDA to issue Internet warnings regarding the risks of vaping (8). While the FDA has serious concerns regarding their use, Health New Zealand Ltd. (HNZ), a private enterprise which analyzed the safety of the Ruyan^®^ e-cigarette with Ruyan^®^ financial support, recommends the use of e-cigarettes as an alternative to traditional smoking (9). HNZ bases its recommendation on the likelihood that vaping is potentially less dangerous than traditional smoking; in other words, their message is “harm reduction.” Cahn and Siegel (10) support HNZ’s recommendation, concluding that “electronic cigarettes show tremendous promise in the fight against tobacco-related morbidity and mortality.” In addition to reducing tobacco-related morbidity and mortality, vaping may also reduce harm incurred from second hand smoke and benefit the environment (11).

Foulds et al. (12, 13) believe that more research needs to be conducted to determine the safety and efficacy of e-cigarettes as a smoking cessation tool. However, they also state that individuals who have successfully quit smoking in favor of vaping should continue to use e-cigarettes as a healthier alternative to conventional cigarettes. Although there are clear perceptions among e-cigarette users that e-cigarettes can be used as both a smoking cessation tool, and a safer alternative to smoking, they can be marketed as neither. The FDCA (5) precludes their use as a smoking cessation tool, and the Family Smoking Prevention and Tobacco Control Act (14) precludes their use as a reduced-risk alternative; therefore, e-cigarettes must be sold as a tobacco product. E-cigarettes could play an important role in the future of smoking cessation, but their use is currently clouded by a tangle of legal and political issues. It is evident that more research on the safety and efficacy of e-cigarettes needs to be conducted, and that more stringent quality control measures should be implemented in order for the legal and political ramifications surrounding these products can be untangled.

The medical community must prepare itself to face the new challenge concerning e-cigarettes and vaping as a “harm reduction” tool. As a consequence of past lessons learned from “Big Tobacco” companies, the medical community is suspicious of e-cigarettes and has routinely advised against their use (15). The medical community advises on the side of caution, indicating that very little scientific evidence is available to show, one way or the other, that e-cigarettes are safe to use, or that they help in the smoking cessation process. In addition, many physicians fear that patients who vape are merely substituting one form of nicotine addiction for another. While there are certainly potential perils associated with vaping, smoking, the leading cause of preventable disease in the United States, is likely to be more dangerous than vaping, especially when considering the myriad of known toxins found in cigarette smoke and the diseases which they promote. Assuming this premise is true, what should the primary medical focus be for a patient who has successfully transitioned from conventional cigarettes to e-cigarettes? Should it be to maintain smoking abstinence, or should it be to quit vaping? Would it not be prudent for a patient who is unwilling to quit smoking or give up nicotine to vape instead of smoke? Given these circumstances, how should patients be advised? The potential health hazards of nicotine addiction from smokeless tobacco products have previously been reviewed in a policy statement by the American Heart Association and include hemodynamic effects, endothelial dysfunction, thrombogenesis, systemic inflammation, and other metabolic effects (16). Understandably, the medical community (15) is concerned that increased availability of e-cigarettes could increase worldwide nicotine dependence, especially among the young as they are enticed by the various flavor options e-cigarettes have to offer. Since vaping does not produce smoke from burning tobacco, the opponents of e-cigarettes fear that traditional smokers will substitute vaping for smoking in settings where smoking is not permitted without any real intention of quitting conventional cigarettes. Furthermore, vaping in public places, coupled with recent e-cigarette commercials on national television, could possibly undermine or weaken current antismoking regulations. Health care professionals will need to consider and weigh what is more harmful to the public, continued smoking or increased nicotine addiction. As e-cigarettes gain greater popularity among smokers, these challenges will undoubtedly occur with increasing frequency.

In order to face this new challenge, health care professionals will need to become familiar with the available scientific evidence-based literature concerning e-cigarettes and vaping. Currently, this literature is sparse, but growing fast, and primarily deals with (1) consumer-based surveys regarding personal views on vaping, (2) chemical analysis of e-cigarette cartridges, solutions, and mist, (3) nicotine content, delivery, and pharmacokinetics, and (4) clinical and physiological studies investigating the acute effects of vaping. Only after reviewing the current literature can physicians and other health care providers give appropriate counsel regarding the role of e-cigarettes and vaping as a safer alternative to smoking, and as a smoking cessation tool (17). Consequently, the aim of this article is to provide a review of the current literature concerning e-cigarettes and vaping so that the medical community can better prepare for the new challenge these devices bring to clinical medicine and public health. The search for relevant scientific literature was accomplished using the pubmed database in which the key words electronic cigarette(s) or e-cigarette(s) were used. The search for articles extended back to 2008 and only highly relevant evidence-based primary literature was retrieved for review. Sixty-six articles dealing with surveys soliciting personal views on vaping; studies analyzing potential toxins and contaminants in e-cigarette cartridges, solutions, and mist; reports profiling nicotine content, delivery, and pharmacokinetics; and clinical and physiological studies investigating the effects of acute vaping were ultimately used. Of these articles, two were published between 2008 and 2009. Six, ten, and fifteen articles were published in 2010, 2011, and 2012, respectively. Thus far, 33 highly relevant articles have been published in 2013, indicating a progressive increase in e-cigarette related research.

## Consumer-Based Surveys

Surveys have shown that awareness of e-cigarettes has quadrupled between 2009 and 2011 (18) and that they have a high adoption rate among traditional smokers (19, 20). Many current and ex-smokers use e-cigarettes as a nicotine replacement therapy (NRT) to help them reduce or quit smoking (13, 21–25) while others use e-cigarettes as a less harmful alternative to smoking (21, 23, 26, 27). At the end of 6 months, Polosa et al. found that vaping e-cigarettes decreased consumption of conventional cigarettes by 80% after 6 months (28) and 50% after 24 months (29). Caponnetto et al. reported similar reductions in cigarette consumption and cigarette abstinence after a year-long trial of using e-cigarettes in both normal smokers (30) and in chronic schizophrenic smokers (31). The authors claim that withdrawal symptoms were minimal and that the perception and acceptance of e-cigarettes was satisfactory, even in the schizophrenic patients. The results of Vickerman et al. (32) are less optimistic. They reported that nearly a third of 2758 callers to six state tobacco quit lines had ever used e-cigarettes of which 61.7% used the e-cigarettes for <1 month. Barbeau et al. (26) reported that using e-cigarettes, in comparison to other FDA-approved NRTs, such as nicotine gum, patches, and inhalers, had less annoying side effects and were more effective in preventing relapse, primarily because vaping retained the psychosocial aspects of real smoking better than the FDA-approved NRTs. Hua et al. (33) found a total of 405 different health-related effects (78 positive, 326 negative, and 1 neutral) reported by e-cigarette users in three different online forums. Users reporting negative health-related effects often reported multiple symptoms, while users reporting positive health-related effects usually reported a single symptom. Additionally, negative health-related effects occurred most frequently in the respiratory, neurological, sensory, and digestive systems while the positive health-related effects occurred solely in the respiratory system.

It is possible that the decreased daily consumption of conventional cigarettes among e-cigarette users, as seen in some studies (28–31), is at least partially due to a psychological element involving smokers’ motivation to quit. Support for this idea is seen in a recent Hawaiian multiethnic study (34) involving 1567 traditional smokers of which 13% were also e-cigarette users attempting to quit smoking. This survey reported that smokers who used e-cigarettes as a smoking cessation tool were more serious about wanting to quit smoking as compared to smokers who did not use e-cigarettes. In addition, e-cigarettes were also viewed as a viable option to other FDA-approved smoking cessation tools. Sutfin et al. (35) surveyed 4444 students from eight North Carolina colleges and found that 216 of these students had experimented with e-cigarettes (ever e-cigarette users) while 4228 had never used e-cigarettes (never e-cigarette users). Of the ever e-cigarette users, 12% were never smokers, 30% were former or experimental smokers, 33% were current non-daily smokers, and 9% were current daily smokers compared to 53, 19, 14, and 4%, for the never e-cigarette users, respectively. When the ever e-cigarette users were asked about e-cigarette harm perception, 17% indicated e-cigarettes are as harmful as conventional cigarettes, 45% responded with less harmful, 3% thought e-cigarettes to be more harmful, and 23% were unsure. The never e-cigarette users responded with 16, 22, 2, and 51%, respectively. This data suggest that vaping is more common, but not exclusive, among traditional smokers. Another statistic revealed that vaping among young college students (mean age 20.7 ± 2.9 years) does not appear to be motivated by any intention to quit smoking. This is somewhat in contrast to Pokhrel et al. (34) who indicated more serious intentions toward smoking cessation among an older population of smokers using e-cigarettes (mean age 42.3 ± 1.02 years) compared to smokers not using e-cigarettes (mean age 45.63 ± 0.35 years). They also reported that individuals who took up vaping as a means to quit smoking were significantly younger and had smoked for less years than those who never vaped.

A concern of the FDA (8) and the medical community (15) is that availability of e-cigarettes will entice teens and young adults toward vaping, which could ultimately lead to smoking conventional cigarettes. Currently, there is little or no concrete evidence confirming the validity of this concern. Cho et al. (36) used data collected from a Korean Health Project to determine awareness and use of e-cigarettes. They found that 10.2% of 4353 students were aware of e-cigarettes, but only 0.5% of those students had actually tried e-cigarettes. Pepper et al. (37) conducted a national online survey of 228 male adolescents (ages 11–19) and determined that <1% of these individuals actually tried e-cigarettes. On the other hand, 67% of the respondents were aware of e-cigarettes with awareness being higher among the older boys. Of those individuals who never tried e-cigarettes, 18% were willing to experiment with no preference toward flavored versus unflavored e-cigarettes. Additionally, smokers were more amiable to experiment with e-cigarettes than non-smokers. In contrast, discussions with 11 focus groups involving 66 young adults (ages 18–26) revealed that young adults favorably perceive e-cigarettes and other new tobacco products specifically because they come in different flavors and that eliminating these flavors may reduce intentions to try these products (38). Another study surveyed 2624 US Midwestern young adults (ages 20–28) and indicated that 69.9% of the respondents were aware of e-cigarettes, but that only 7% actually tried vaping (39). Goniewicz et al. (40) conducted a survey of students enrolled at 176 nationally representative Polish high schools (ages 15–19) and universities (ages 20–24) and reported that 23.5% of high school students and 19% of university students had ever tried e-cigarettes. Of all the students who tried e-cigarettes, only 3.2% were non-smokers, which compares closely to the 4.9% reported by Sutfin et al. (35). Other strong correlates of e-cigarette use among adolescents include male gender, and having parents who smoke (36, 40). While a small percentage of young non-smokers experiment with e-cigarettes, it is more likely that young smokers will experiment with e-cigarettes. One fact emerges from these studies; as e-cigarette popularity increases, so does awareness of them among young individuals. How increasing awareness will ultimately affect e-cigarette usage by adolescent and young adults remains to be seen.

A number of studies (41–43) indicate that all forms of NRT are at least initially successful in maintaining cigarette abstinence. However, the successful long-term smoking cessation rate still remains relatively low. Employing a meta-analysis study, Hughes et al. (41) found the 6-month smoking quit rates for NRTs to be between 1 and 11% in seven studies as compared to between 3 and 5% in smokers who tried to quit on their own (44). Rennard et al. (42) reported a quit rate of 8% among smokers who used the nicotine inhaler for 15 months. In contrast, few studies have tested e-cigarettes as a smoking cessation tool (28, 29, 45). From an online survey, Siegal et al. (45) reported that 31% (69 of 216) of the respondents were no longer smoking cigarettes after 6 months of using e-cigarettes. Of those respondents who quit smoking, 57% were still using e-cigarettes, 9% were using other tobacco free nicotine products, and 34% were completely nicotine free. Polosa et al. (28) investigated the effect of e-cigarettes on smoking cessation and discovered that 22.5% (9 of 40) of the participants had not had a cigarette in 6 months. Of that cohort, 67% were still using e-cigarettes while 33% were nicotine free. Similar results were reported in a 24-month study by Polosa et al. (29). These studies and others (46, 47) suggest that e-cigarettes could play a role in smoking reduction and cessation, and as a result could reduce the harm incurred by smoking as effectively as any FDA-approved NRT. However, the role of e-cigarettes on total nicotine abstinence is still highly questionable, and it has been suggested that one form of nicotine addiction is simply replacing another (26). A summary of the studies involving consumer-based surveys regarding personal views of vaping are shown in Table 1.

**Table 1:** **Studies involving consumer-based surveys regarding personal views on vaping**.

Authors (Reference)	Study design	Participants	Participant’s location	Key finding
**STUDIES REPORTING POSITIVE OR NEUTRAL IMPACT OF E-CIGARETTES, VAPING, OR HARM REDUCTION**				
Etter (21)	Online French survey at www.StopTabac.ch	81 Respondents, ages 19–65, 77% male, 63% former smokers, 23% daily smokers, 13% occasional smokers	81% France	E-cigarettes were used to quit smoking
			8% Belgium	
			6% Canada	
			5% Switzerland	
Cho et al. (36)	Survey of adolescents from five schools participating in a 2008 Health Promotion Fund Project	4353 Adolescent students	Korea	444 adolescent students had heard of e-cigarettes. 22 adolescents had ever tried e-cigarettes. Significant predictors of e-cigarette use include male gender and cigarette smoking experience
Etter and Bullen (22)	Survey of visitors to websites and online discussion forums	3587 Respondents, ages 31–52, 63% male, 70% former smokers, 19% daily smokers, 11% occasional smokers	62% United States	E-cigarette users believe that e-cigarettes helped them to quit or reduce smoking, and that vaping is less toxic than smoking
			14% France	
			6% United Kingdom	
			4% Switzerland	
			3% Canada	
Foulds et al. (12)	Survey conducted at e-cigarette enthusiast’s convention	104 Respondents, mean age 34 ± 9, 74% male, 88% Caucasian, 78% former smokers, 19% daily smokers	Philly Vapefest, 2011, Philadelphia, PA	E-cigarette users believe that e-cigarettes helped them to quit smoking, and vaping is less harmful than smoking
Polosa et al. (28)	Tracking daily consumption of cigarettes for 6 months	40 Subjects (26 males) began and 27 subjects (18 males) ended 6 months later	Recruited from the local hospital staff in Catania, Italy	Vaping e-cigarettes decreased consumption of traditional cigarettes as verified by exhaled carbon monoxide
		Mean age 43 ± 9 years	
		Years smoked 27 ± 9 years	
Siegal et al. (45)	Online survey of new e-cigarette users who recently purchased Blue e-cigarettes	216 Respondents (72% male), ages 18–65+ years, smokers for 5–30+ years	Worldwide. E-mail addresses were provided for 5000 first time online e-cigarette purchasers from the Blue-cigarette distributor and served as the pool of respondents	After 6 months of vaping, 31% of respondents were no longer smoking cigarettes
Goniewicz and Zielinska-Danch (40)	Survey of students enrolled in at 176 nationally representative high schools and universities in Poland	20,240 High school students (15–19 years) and university students (20–24 years) 43% of students were male	Poland	About 20% of polish youth have tried e-cigarettes, but only 3% of never smokers tried e-cigarettes. Not clear if e-cigarettes are a novelty that young people try once. Significant predictors of e-cigarette use include male gender and cigarette smoking experience
Kralikova et al. (19)	Interview of people buying cigarettes between 10 and 19 October 2011	973 Respondents, average age 32 years, and 54% male	Five locations across Prague, Czech Republic	86% of respondents have heard of e-cigarettes
Barbeau et al. (26)	Focus group discussions posing open ended questions	Nine Men and two women, ages 18–64 years	Boston University School of Public Health, Boston, MA, USA	Vaping was more effective in maintaining cigarette abstinence than the FDA-approved nicotine replacement therapies due to retention of behavioral and social components of smoking addiction
Bullen et al. (46)	Survey using a randomized controlled trial. Participants recruited via community newspapers	E-cigarette Group (*n* = 289), age 44 ± 13 years, 38% male. Nicotine patch Group (*n* = 295), age 40 ± 13 years, 38% male. E-cigarette placebo group (*n*-73), 43 ± 12 years, 38% male	Aukland, New Zealand	E-cigarettes, with or without nicotine (placebo e-cigarette), were as effective at helping smokers quit as nicotine patches
Caponnetto et al. (30)	Regular smokers recruited between June 2010 and February 2011 were observed for 12 months	300 Participants, mean age 44 ± 13 years, 63% male and smoke at least 15–25 cigarettes/day	Catania, Italy	The use of e-cigarettes, with or without nicotine, decreased cigarette consumption and elicited tobacco abstinence as verified by exhaled carbon monoxide
Caponnetto et al. (31)	Chronic schizophrenic patients were observed and surveyed for 12 months	14 Schizophrenic patients (6 male), mean age 45 ± 13 years and smoked at least 20–35 cigarettes/day	CTA, Villa Chiara-Psichiatrica Riabilitativa e Ricerca, Catania, Italy	The use of e-cigarettes decreased e-cigarette consumption without causing significant side effects in schizophrenic patients
Choi and Forster (39)	Survey of U.S. Midwestern adults	Cohort of 2624 adults aged 20–28 years	Midwestern United States	Nearly 70% of the respondents were aware of e-cigarettes, and 7% had tried e-cigarettes. Significant predictors of e-cigarette use include male gender and cigarette smoking experience
Dawkins et al. (23)	Online survey hosted by the University of East London with links from TECC/TWEL websites	1347 Respondents, mean age 43 years, 70% male and 96% Caucasian	Respondents from 33 countries (72% European)	E-cigarettes are primarily used for smoking cessation, but for a longer duration than nicotine replacement therapies
Dockrell et al. (24)	Three online surveys and one focus group. Respondents were recruited from panel of adults in Britains	February 2010 online population survey (*n* = 12,597 for all respondents with *n* = 2297 for smokers). April 2010 online smokers’ survey (*n* = 1380). February 2012 online population survey (*n* = 12,432 for all respondents with *n* = 2093 for smokers). March 2010 focus group consisting of smokers (*n* = 37)	Great Britain	E-cigarette use may bridge the smoking cessation process. There is little evidence to suggest that e-cigarettes is widely used among never smokers
Farsalinos et al. (25)	Recruitment and survey of subjects who had completely substituted conventional cigarettes with e-cigarettes for at least 1 month	111 Participants, 84% male, mean age 37 ± 6 years, that smoked at least 20–30, cigarettes/day. Participants had a mean smoking duration of 37 ± 6 years and a smoking cessation duration of 4–11 months	Visitors to a Hospital in Kallithea, Greece and to an electronic cigarette consumers’ internet forum in Greece	E-cigarettes with higher nicotine content were more successful in the smoking cessation process
Goniewicz et al. (27)	Web-based survey of e-cigarette users	179 Respondents	Poland	Participants primarily used e-cigarettes to cease smoking and reduce smoking related harm
Kralikova et al. (20)	Interview of people smoking cigarettes on the street during May of 2012	2012 Respondents, average age 34 years, 51% male	17 cities across the Czech Republic	About one fifth of smokers who try e-cigarettes go on to become regular e-cigarette users
Li et al. (47)	Telephone-based survey by random digit dialing of the New Zealand Smoking Monitor to recruit current smokers and recent quitters	840 Current smokers and recent quitters	New Zealand	Only 7% of respondents ever purchased e-cigarettes, 33% perceived e-cigarettes as less harmful than conventional cigarettes and 41% believed e-cigarettes are an acceptable means to smoking cessation
Pepper et al. (37)	Online national survey of male adolescents in November 2011. Participants recruited through parents who were members of a panel of U.S. households	228 Male adolescents, ages 11–19 years	United States	Only 2 of 228 adolescents had ever tried e-cigarettes.18% of adolescent who were aware of e-cigarettes were also willing to try them with no preference to plain or flavored
Pokhrel et al. (34)	Cross-sectional survey of Hawaiian Islanders recruited through newspaper advertisement from 2010 to 2012	1567 Participants divided into two groups. Ever –e-cigarette users (*n* = 202 students; mean age 42 ± 1 years, 46% male) and never e-cigarette users (*n* = 1365; mean age 46 ± 0.4 years, 51% male)	Hawaiian Islands	Smokers who try e-cigarettes appear to be more serious about smoking cessation and treat e-cigarettes as valid alternatives to FDA-approved nicotine replacement therapies
Polosa et al. (29)	Tracking daily consumption of cigarettes for 24 months	Follow-up observational study of Polosa et al. (28). See above	Recruited from the local hospital staff in Catania, Italy	Long-term e-cigarette use can substantially decrease conventional cigarette consumption as verified by exhaled carbon monoxide
Regan et al. (18)	Consumer-based mail-in survey	10587 Adult (18 years or older) respondents in 2009 and 10328 adult (18 years or older) respondents in 2010	United States	Awareness of e-cigarettes doubled from 16% in 2009 to 32% in 2010
Sutfin et al. (35)	Web-based survey in fall of 2009	4444 Students divided into two groups. Ever e-cigarette users (*n* = 216 students; mean age 21 ± 3 years, 53% male) and never e-cigarette users (*n* = 4228; mean age 21 ± 3 years, 36% male)	Students from eight North Carolina colleges	E-cigarette use is more common among smokers but not exclusive to them. E-cigarette use among college students does not appear to be motivated by intentions to quit
Vickerman et al. (32)	Survey of callers to six state tobacco quit lines 7 months after initially receiving intervention	2758 callers	United States	Only about one third of the respondents had ever tried e-cigarettes of which 62% used for <1 month
			The actual states were not indicated	
**STUDIES REPORTING NEGATIVE IMPACT OF E-CIGARETTES, VAPING, OR HARM REDUCTION**				
Choi et al. (38)	Eleven focus group discussions	66 young adults, ages 18–26 years old	University of Minnesota, Minneapolis, Minnesota	Young adults perceive e-cigarettes and other new tobacco products positively, especially when they are flavored
Hua et al. (33)	Data collected from postings of three high traffic online forums	Total of 632 posts from 560 different posters	Worldwide but mostly from the United States and Canada	A total of 405 different, mostly negative health-related effects, were reported by e-cigarette users

## Chemical Analysis of E-Cigarette Cartridges, Solutions, and Mist

The ingredients found in e-cigarette cartridges and solutions are relatively few, and for the most part non-toxic and non-carcinogenic, especially in the low quantities delivered. They include nicotine, propylene glycol, glycerin, and tobacco flavoring (4, 48). Propylene glycol, an FDA-approved solvent used in foods, a vehicle for intravenous diazepam, and as the major ingredient found in e-cigarette fluids, makes up about 90% of the solution (9). Certain contaminates, most of which are derived from tobacco flavoring, have been detected in e-cigarettes. A small amount of diethylene glycol (approximately 1%), a known carcinogen and an ingredient in anti-freeze, was also detected in one out of 18 cartridges analyzed by the FDA (4). The source of the diethylene glycol contamination is not clear but could reflect the use of non-pharmaceutical grade propylene glycol (10). In comparison, cigarette smoke from burned tobacco products contains thousands of compounds, many of which have been shown to induce or promote carcinogenesis (49); specifically the trace metals (i.e., cadmium, arsenic, chromium, nickel, and lead), the tobacco specific N-nitrosamines (TSNA), the polycyclic aromatic hydrocarbons (PAHs), and the volatile organic compounds (VOCs). While investigations have shown some of these hazardous compounds to be present in e-cigarette cartridges, solutions, and mist, there are only a few reports detecting levels of these contaminates high enough to be of significant risk to humans. The HNZ study (9) found levels of arsenic cadmium, chromium, nickel, and lead to be undetectable in e-cigarette cartridge liquid. In contrast, Williams at al. (50) found levels of lead, chromium, and nickel in e-cigarette aerosol to be equivalent to, and in some cases higher than, what has been reported for cigarette smoke. They indicate that the primary source of these trace metals are the filaments inside the e-cigarette cartomizer (i.e., the aerosolizing component of the e-cigarette), and conclude that improved quality control of e-cigarette design and manufacturing would greatly reduce the presence of these trace metals. The FDA (4) and HNZ (9) both reported that e-cigarettes contain trace amounts of TSNAs, but the levels found in the e-cigarettes represent only a very small fraction (0.008 μg/e-cigarette cartridge containing 16 mg of nicotine) of what is typically found in traditional cigarettes (6.3 μg/full flavor Marlboro cigarette) (51). To put this into perspective, an e-cigarette cartridge is good for about 150–300 puffs while a single conventional cigarette is good for about 10–15 puffs (52). The amount of total TSNAs found in other FDA-approved nicotine products was roughly equivalent to the total amount of TSNAs found in e-cigarettes (9, 10). Other studies (11, 53–56) confirm the low levels of TSNAs present in e-cigarette solutions and vapor, as well as the low or undetectable levels of particulate matter, trace metals, VOCs, and PAHs, especially when compared to the amounts present in cigarette smoke.

As previously mentioned (2), the FDA issued warnings to several e-cigarette companies for selling e-cartridges and refill solutions containing active pharmaceutical ingredients such as rimonabant (Zimulti^®^) for the purpose of losing weight and reducing smoking addiction, and tadalafil (the active ingredient in Cialis^®^) for the purpose of increasing sexual capacity. FDA analyses of these e-cartridges and solutions revealed the presence of amino-tadalafil and not tadalafil, and the presence of an oxidative product of rimonabant, as well as rimonabant (57), although the amount of either of these substances that is able to transfer from liquid to vapor phase is low (58). Table 2 summarizes the studies involving chemical analyses of e-cigarette cartridges, solutions, and mist.

**Table 2:** **Studies involving chemical analysis of e-cigarette cartridges, solutions, and mist**.

Authors (Reference)	E-cigarette brand	Substances tested	Analysis	Key finding
**STUDIES REPORTING POSITIVE OR NEUTRAL IMPACT OF E-CIGARETTES, VAPING, OR HARM REDUCTION BASED ON THE ABSENCE OR PRESENCE OF SPECIFIC TOXINS**				
Laugesen (9)	Runyon	TSNA	LC-MS	TSNAs are present but at levels much lower than in conventional cigarettes and too small to be carcinogenic
*Research funded by Runyan*		MAO-A and B inhibitors	Flourometric assay	MAO-A and B are inhibited by tobacco smoke but unaffected by e-cigarette fluid
		PAH	GS-MS	Polycyclic aromatic hydrocarbons undetectable
		Heavy metals	ICP-MS	Heavy metals were undetectable
		CO	CO analyzer	Exhaled carbon monoxide does not increase after e-cigarette use
McAuley et al. (11)	Brand not indicated.	TSNA	GC/MS	TSNA, PAH, diethylene glycol, VOC, and carbonyls in e-cigarette mist were all negligible compared to cigarette smoke.
		PAH	GC/MS	
		Diethylene Glycol	GC/MS	
		VOC	HS-GC/MS	
		Carbonyls	HPLC-UV	
Pellegrino et. al. (56)	Italian brand of e-cigarettes	Particulate matter	Particle counter and smoking machine	Particulate matter is lower in e-cigarette mist compared to cigarette smoke
	Abstract in English	
	Full article in Italian	
Goniewicz et al. (53)	Eleven brands of Polish and one brand of English e-cigarettes	CarbonylsVOCTSNAHeavy metals	HPLC-DAD GC-MS UPLC-MS ICP-MS	TSNA, VOC, and carbonyl compounds were determined to be between 9 and 450 times lower in e-cigarettes mist compared to conventional cigarette smoke
				Heavy metals present in e-cigarette mist
Kim and Shin (55)	105 Replacement liquid brands from 11 Korean e-cigarette companies	TSNA	LC-MS	TSNAs are present at low levels in e-cigarette replacement liquids
Schripp et al. (54)	Three unidentified brands	VOC	GC-MS	VOC in e-cigarette cartridges, solutions, and aerosolized mist were low or undetectable compared to conventional cigarettes
		Particulate matter	Particle counter and smoking machine	Particulate matter is lower in e-cigarette mist compared to cigarette smoke
**STUDIES REPORTING NEGATIVE IMPACT OF E-CIGARETTES, VAPING, OR HARM REDUCTION BASED ON THE PRESENCE OF SPECIFIC TOXINS**				
Westenberger (4)	Njoy	TSNA	LC-MS	TSNA present
*FDA study*	Smoking everywhere	Diethylene glycol	GC-MS	Diethylene glycol present
		Tobacco specific impurities	GC-MS	Tobacco specific impurities present
Trehy et al. (58) *FDA study*	Njoy	Nicotine related impurities	HPLC-DAD	Nicotine related impurities present
	Smoking everywhere	
	CIXI	
	Johnson creek	
Hadwiger et al. (57) *FDA study*	Brand not indicated	Amino-tadalafil	HPLC-DAD-MMI-MS	Amino-tadalafil present
		Rimonabant		Rimonabant present
Williams et al. (50)	Brand not indicated	Heavy metals	ICP-MS	Heavy metal and silicate particles present in e-cigarette mist
		Silicate particles	Particle counter and smoking machine, light and electron microscopy, cytotoxicity testing, x-ray microanalysis	

TSNA, tobacco specific nitrosoamines; LC-MS, liquid chromatography-mass spectrometry; MAO-A and B, monoamineoxidase A and B; PAH, polycyclic aromatic hydrocarbons; GS-MS, gas chromatography – mass spectrometry; ICP-MS, inductively coupled plasma – mass spectrometry; CO, carbon monoxide, VOC, volatile organic compounds; UPLC-MS, ultra-performance liquid chromatography-mass spectrometry; HPLC-DAD-MMI-MS, high performance liquid chromatography-diode array detector-multi-mode ionization-mass spectrometry.

## Nicotine Content, Delivery, and Pharmacokinetics

E-cigarettes are designed to deliver nicotine in an aerosolized manner that simulates an authentic smoking experience without the real smoke. In this respect, e-cigarettes are similar to the FDA-approved nicotine inhaler. Bullen et al. (59) determined the Ruyan^®^ e-cigarette had a nicotine pharmacokinetic profile very similar to the Nicotrol^®^ inhaler, but the study’s participants thought the e-cigarettes were more pleasant to use and produced less irritation to the mouth and throat. For e-cigarettes, the nicotine is delivered through cartridges prefilled with a nicotine solution or cartridges that the user fills with a nicotine refill solution. In either case, the nicotine concentration of the solutions or cartridges can be purchased in strengths ranging from 0 to 24 mg or more, according to user preference. Unfortunately, the amounts of nicotine specified on the labels of various brands of e-cartridges and solutions have not always been accurate or consistent (60). The FDA (4, 57, 58) confirmed the ability of e-cigarettes to deliver nicotine, but stated there is too much variability in the amount of nicotine delivered per puff of any e-cigarette cartridge for them to be considered safe. Repeated analysis of a menthol high strength Njoy^®^ e-cigarette cartridge (18 mg of nicotine) yielded nicotine deliveries of 26.8, 34.9, and 43.2 μg/100 ml puff. The medium strength Smoking Everywhere^®^ e-cigarette cartridge (11 mg of nicotine) and the medium strength Njoy^®^ e-cigarette cartridge (12 mg of nicotine) delivered 15.7 and 10.6 μg nicotine/100 ml puff, respectively, and were found to be similar to the 10-mg Nicotrol^®^ inhaler shown to deliver 15.2 μg nicotine/100 ml puff. Of major concern is that some e-cartridges and solutions that were labeled as containing 0 mg of nicotine did in fact contain some nicotine (4, 57). The FDA (4, 58) also detected small quantities of cotinine, a metabolite of nicotine, and several nicotine related impurities to include, anabasine, anatabine, myosmine, and β-nicotyrine in some, but not all, e-cartridge solutions and mist samples analyzed. Flouris and Oikonomou (61) question the rigor by which the FDA conducted these analyses, indicating that these analyses “cannot be used to draw conclusions or inferences about potential effects on health” until more rigorous chemical analyses, followed by extensive animal and clinical trials in humans, are conducted. Two other studies have also found discrepancies in the labeled nicotine content compared to the actual nicotine content in a number of e-cigarette brands (52, 62). Goniewicz et al. (52) reported relative percent differences between the labeled and actual nicotine concentration per cartridges (or refill fluids) to range between −89 and 28% in 30 popular brands of e-cigarettes. Cameron et al. (62) found the actual nicotine concentration in e-cigarette cartridges and refill fluids to range from 1.8 to 23.7 mg/ml less than the labeled nicotine concentration. However, a more recent study analyzing several brands of e-cigarette refill solutions did find nicotine content to be accurate and consistent to what was printed on the label (63). Inconsistencies reported in nicotine concentrations, and hence deliveries, could be a reason why some e-cigarette users and not others report adverse reactions such as mouth and throat irritation, vertigo, headache, and nausea (21, 26, 33). Incidentally, some of these same adverse reactions have also been reported for various FDA-approved NRTs (64). In a study evaluating design features, accuracy and clarity of labeling, and quality of printed materials and instruction manuals for e-cigarettes it was concluded that design flaws, inadequate product labeling, and lack of quality control in the manufacturing of e-cigarettes are an indication that stricter oversight and regulation are required for these devices (65). Accurate production, safe packaging, and proper storage of e-cigarette refill solutions are critical. Typically, a 5-ml vial of e-cigarette refill solution could contain a nicotine concentration of 20 mg/ml or 100 mg/vial, and the known lethal dose of nicotine has been estimated to be about 10 mg in children and between 30 and 60 mg in adults (62). Given the potential health hazards of nicotine (16), inadvertent skin contact, or consumption of just one of these vials by children or pets could have tragic consequences. It is important that extreme caution be used when storing nicotine solutions.

Regardless of the inaccuracies and inconsistencies in the production of e-cigarette cartridges and solutions, puff-for-puff, the amount of nicotine finding its way into the blood stream from vaping an e-cigarette has been shown to be less than what you would expect from smoking a conventional cigarette with comparable nicotine content (59, 66, 67). These studies report little or no increases in blood nicotine levels of naive subjects after acute predefined use of e-cigarettes compared to conventional cigarettes. According to Bullen et al. (59), serum levels of nicotine were similar after use of either the Nicotrol^®^ inhaler or a Ruyan^®^ e-cigarette. They found serum nicotine levels to peak at 1.3 ng/ml after 19.6 min of vaping an e-cigarette, and 2.1 ng/ml after 32 min of using the Nicotrol^®^ inhaler, compared to 13.4 ng/ml after 14.3 min of smoking a cigarette. The Nicotrol^®^ inhaler is said to be inappropriately named since it does not deliver significant quantities of nicotine directly to the lungs (68, 69). This is because the particle size of the delivered nicotine is too large to effectively reach pulmonary alveoli (70, 71). With each puff, the inhaler delivers nicotine to the oral cavity which is subsequently absorbed by the buccal mucosa and pharyngeal mucosa. It is not clear where most of the nicotine from e-cigarettes is primarily absorbed; the alveoli, the airways or the oral cavity. *In vitro* evidence suggests that e-cigarette aerosol particle size and distribution in the respiratory system is similar to conventional cigarette smoke (72–74). Sahu et al. (72) found the particle size of mainstream cigarette smoke to range between 186 and 198 nm and deliveries to the pulmonary alveoli, tracheal, and bronchiolar airways, and oral cavity were predicted to be 29.8, 15.2, and 16.3%, respectively. Zhang et al. (73) determined average e-cigarette aerosol particle diameters to be approximately 400 nm, and alveolar deliveries to be between 7 and 18%. Zhang et al. (73) also indicate that nicotine delivery is highly dependent on a number of factors, including vaping technique, particle evolution, and cloud effects. It is very likely that aerosol particle size and nicotine delivery via e-cigarettes may have similar distribution profiles that are intermediate between conventional cigarettes and the Nicotrol^®^ inhaler.

In contrast to the aforementioned investigations (59, 66, 67), three other study (75–77) found increased blood levels of nicotine in experienced e-cigarette users within 5 min of the first puff of an e-cigarette. Dawkins et al. (76) reported blood nicotine levels to increase from 0.74 ng/ml baseline to 6.77 ng/ml 10 min after 10 puffs of an e-cigarette, achieving a mean 13.91 ng/ml by the end of a 1-h *ad libitum* vaping period. It has also been reported that cotinine in the saliva (78) and serum (79) of e-cigarette users is significantly elevated to levels commonly found in cigarette smokers. Vansickel and Eissenberg (75) also reported an increase in heart rate, which is not surprising since smoking and nicotine have long been known to stimulate heart rate and blood pressure (80, 81). It is interesting to note that the 2010 Vansickel et al. (67) study, and in Czogala et al. (82), heart rate and nicotine levels were significantly increased in smokers, but not vapers. However, the 2013 Vansickel and Eissenberg study (75) reported that both smoking and vaping-induced similar concomitant increases in heart rate and blood levels of nicotine. As suggested by Farsalinos et al. (83), this discrepancy could be attributed to differences in experimental design, and puffing topography of the participants in each study (i.e., different daily durations of vaping, experience with e-cigarette devices, personal puffing characteristics to include the amount of vacuum created on every puff, and the vaping-induced deposition of nicotine into the oral cavity and/or size of the aerosolized particles). Trtchounian et al. (84), determined that smoke/aerosol density remained fairly constant while puffing on a conventional cigarette from start to finish (approximately 10 puffs), although variations did exist between brands of conventional cigarettes. The aerosol density for e-cigarettes, while higher than conventional cigarettes in three out of the four brands tested, also remained fairly constant for the first 10 puffs of a new e-cigarette cartridge. However, a decremental decrease in aerosol density was observed as each cartridge approached its terminal life. Consequently, this decrease in aerosol density would require the person vaping to generate more vacuum to maintain an aerosol density equivalent to the initial puffs and could be a reason contributing to longer puff duration for electronic cigarettes than for conventional cigarettes (85). Similar variations in the rate of airflow required to produce aerosol between and within brands of e-cigarettes were also reported by Williams and Talbot (86). According to Goniewicz et al. (52), these studies demonstrate the importance of the initial nicotine content, the efficiency of the vaporization process that determines how much of the nicotine gets aerosolized, and the individual’s puffing topography on the efficacy of nicotine delivery from e-cigarettes.

Many smokers claim that smoking cigarettes increases cognitive awareness, reduces stress, and induces a pleasurable feeling of wellbeing. Consequently, this is what makes smoking cigarettes so enjoyable and addictive. It is suggested that smoking has some psychological beneficial effects relating to job performance, vigilance, and mnemonic tasks, and that these effects are induced by nicotine, the addictive ingredient in tobacco (87). Similar effects have also been noted in non-smokers after a single dose of nicotine (88), and it is also worth mentioning that nicotine may have an ameliorating effect on both Parkinson’s and Alzheimer’s patients (89). Dawkins et al. (90, 91) found a decrease in the desire to smoke and reduced withdrawal symptoms associated with tobacco abstinence (1–10 h) among smokers vaping e-cigarettes with nicotine in comparison to e-cigarettes without nicotine. Furthermore, the nicotine from the e-cigarette also improved prospective memory and working memory performance. Nicotine is a central nervous system (CNS) stimulant, and as such it is possible that a psychological need to enhance cognitive functioning reinforces addiction in smokers (92). Nicotine is also known to stimulate adrenergic and dopaminergic neurons in mesolimbic areas of the brain involved with reinforcing pleasurable reward behavior (93). Monoamine oxidases (MAO) normally reduce nicotine-induced adrenergic and dopaminergic activities by oxidizing them to inactive metabolites, and thereby limiting reward behavior. For cigarette smokers, however, nicotine is made even more addictive by synergizing with MAO inhibitors known to be present in cigarette smoke (94). Supporting evidence has been shown by Fowler et al. (95, 96) in which the activities of both MAO-A and MAO-B were reduced in various brain regions of smokers but not of non-smokers. Lewis et al. (94) indicate that there are at least six different MAO inhibitors present in cigarette smoke. In contrast, Laugesen et al. (9) were unable to detect any MAO inhibitors in e-cigarette cartridges or the inhaled aerosol mist. These studies suggest that nicotine from e-cigarettes and other FDA-approved NRTs may be less addictive than nicotine from burned tobacco products, and may be the reason why e-cigarette users report a suppression of smoking and nicotine cravings (59, 66, 67, 90, 91). These investigations support the rationale behind NRT treatment for smoking cessation, which is that nicotine from NRTs, and possibly e-cigarettes, does not occupy the nicotinic receptors to the same extent as nicotine from tobacco smoke (97). The effect is reducing withdrawal symptoms and cravings for cigarettes (71, 98) while possibly still providing some enhanced cognitive awareness and pleasurable reward (92, 93). A summary of the studies involving nicotine content, delivery, and pharmacokinetics are listed in Table 3.

**Table 3:** **Studies involving nicotine content, delivery, and pharmacokinetics**.

Authors (Reference)	E-cigarette brand	Devices, substances or parameters tested	Study design and analysis	Key finding
**STUDIES REPORTING POSITIVE OR NEUTRAL IMPACT OF E-CIGARETTES AND VAPING AS COMPARED TO CONVENTIONAL CIGARETTES AND SMOKING**				
Bullen et al. (59)	Ruyan	Nicotine pharmacokinetic profile was determined in …	Plasma nicotine (ng/ml) was determined by HPLC-EC in participants who smoked at least 10 cigarettes/day	Vaping e-cigarettes produce a nicotine pharmacokinetic profile very similar to the Nicotrol inhalers but considerably lower than smoking a cigarette
		E-cigarette with 16 mg nicotine (*n* = 8)	
		Nicotrol inhaler with 10 mg nicotine (*n* = 10)	
		Participant’s usual cigarette (*n* = 9)	
Eissenberg (66)	Njoy Crown seven	Plasma nicotine and heart rate measured before and after 10 puffs of each device was determined in the following groups …	Sixteen smokers, naïve to e-cigarettes were cycled through the four device groups. Participants were required to have a 12-h period of cigarette abstinence before the start of each device test and 48-h between each device test	Smoking, but not vaping, significantly increased plasma nicotine and heart rate
		Njoy (16 mg nicotine)	
		Crown seven (16 mg nicotine) own brand cigarette	
		Sham own brand cigarette	
Vansickel et al. (67)	Njoy Crown seven	Plasma nicotine, expired carbon monoxide and heart rate measured before and after 10 puffs of each device were determined in the following groups …	Sixteen smokers, naïve to e-cigarettes were cycled through the four device groups. Participants were required to have a 12-h period of cigarette abstinence before the start of each device test and 48-h between each device test	Plasma levels of nicotine, expired carbon monoxide, and heart rate all increased after smoking, but not vaping
		Njoy (18 mg nicotine)	
		Crown seven (16 mg nicotine)	
		Own brand cigarette	
		Sham own brand cigarette	
Etter and Bullen (78)	Own brand e-cigarettes	Salivary cotinine and heart rate	Experienced e-cigarette users vaped *ad libitum* but abstained from cigarettes or NRTs for 48 h upon which salivary cotinine was collected and heart rate determined	Vaping and smoking induce similar increases in salivary cotinine levels and heart rate
Czogala et al. (82)	Brand not indicated Abstract in English Full article In Polish	Systolic pressure Diastolic pressure Pulse Heart rate	Comparison of hemodynamic parameters in smokers (*n* = 42; 50% male) after smoking a cigarette or vaping an e-cigarette	Vaping e-cigarettes failed to induce the typical hemodynamic parameters associated with traditional smoking
Dawkins et al. (91)	White Super	Desire to smoke Nicotine withdrawal symptoms Attention and working memory was determined in the following groups … E-cigarette (18 mg nicotine) E-cigarette (0 mg nicotine) Just hold e-cigarette	Random allocation of 86 smokers into one of three groups. Desire to smoke and withdrawal symptoms rated at 0, 5, and 20 min after vaping *ad libitum* for 5 min. Attention and working memory was determined using “The Letter Cancelation” and “Brown–Peterson Working Memory” tests	E-cigarettes eliminated nicotine withdrawal symptoms and desire to smoke and enhanced working memory performance, suggesting efficient nicotine delivery
Ingebrethesen et al. (74)	Two different brands not specified	E-cigarette aerosol particle size	Smoking machine and spectral transmission procedure	Particle size in e-cigarette aerosol and conventional cigarette smoke for nicotine delivery are similar
		Cigarette smoke particle size	
Vansickel et al. (77)	Vapor king	Plasma nicotine concentration, heart rate, urge to smoke cigarette, and nicotine withdrawal symptoms tested in four sessions. Session 1: 10 puffs of e-cigarette; Session 2: choice of 10 puffs of e-cigarette or cash; Session 3: choice of 10 puffs of e-cigarette or 10 puffs of conventional cigarette; Session 4: choice of 10 puffs of conventional cigarette or cash. Each session consisted of 6 bouts of puffing and bouts were 30 min apart	Twenty smokers, not currently using e-cigarettes, were cycled through four experimental sessions. Participants were required to have a 12-h period of cigarette abstinence before the start of each device test and 48-h between each device test No mention of how nicotine was assayed	E-cigarettes deliver significant amounts of nicotine, increase heart rate, and reduce nicotine withdrawal symptoms and the urge to smoke
Zhang et al. (73)	Bloog MaxX fusion	E-cigarette aerosol particle size Alveolar delivery	Smoking machine and scanning mobility particle sizer	E-cigarette aerosol particle diameter was slightly larger and calculated alveolar delivery is slightly lower when compared to cigarette smoke. Nicotine delivery depends on vaping technique, particle evolution, and cloud effect
Dawkins and Corcoran (76)	First-generation e-cigarette (18 mg/ml nicotine)	Plasma nicotine Tobacco withdrawal symptoms Urge to smoke	Fourteen experienced e-cigarette users abstinent from smoking and vaping for 12 h before test period	Vaping-induced reliable nicotine delivery after acute use in experienced e-cigarette users. Tobacco-related withdrawal symptoms and urge to smoke were reduced
			Blood samples collected at baseline (0 min), after 10 puffs, after 1 h *ad libitum*, and after a 2-h rest period	
Dawkins et al. (90)	Tornado	Desire to smoke and prospective memory was determined in two sessions	Twenty smokers, abstinent for 8–10 h, cycled through two experimental sessions. Desire to smoke was determined using “Single-Item Desire to Smoke Scale” and “Mood and Physical Symptoms Scale.” Prospective memory was measured using “Cambridge Prospective Memory Test”	Nicotine from e-cigarettes improves time-based prospective memory, suggesting efficient nicotine delivery
		Session 1: e-cigarette (18 mg nicotine)	
		Session 2: e-cigarette (0 mg nicotine)	
Etter et al. (63)	Twenty models of the 10 most popular brands of refill liquids for e-cigarettes	Contents of nicotine, nicotine degradation products, and nicotine impurities	GC and LC	The nicotine content in refill bottles are close to what is indicated on the label. Impurities in several brands are detectable but at levels considered harmless
Farsalinos et al. (83)	Nobacco	Puff number and duration, inhalation time, exhalation time, and nicotine consumed was determined in the following groups …	Forty-five e-cigarette users and 35 smokers (smokers were in a randomized cross-over design) were observed for puff number and duration, and inhalation and exhalation times using video recordings. Nicotine consumed (e-cigarette group only) was measured by loss of weight of liquid in cartridge using a precision balance	E-cigarette use topography and conventional cigarette use topography are different. At least 20 mg/ml nicotine in e-cigarette liquid is required to deliver the same amount of nicotine as conventional cigarettes
		E-cigarette users (vaping)	
		Smokers (smoking and vaping subgroups)	
Flouris et al. (79)	Nobacco	Serum cotinine was determined in … Smokers Never smokers	Smokers (*n* = 15) went through a control session, an active smoking session, and an active vaping session. Never smokers went through control, passive smoking and passive vaping sessions. LC-MC used to measure serum cotinine	Acute vaping and acute smoking induce similar increases in serum cotinine levels
Goniewicz et al. (52)	Sixteen popular brands of e-cigarettes from Poland, United Kingdom, and United States	Nicotine content in e-cigarette aerosol	E-cigarette aerosol was generated using smoking machine. Nicotine content in aerosol was determined using GS-TSD	Nicotine in e-cigarette aerosol is lower than in cigarette smoke. Efficacy and consistency of nicotine vaporization between brands is variable
Hua et al. (85)	Various brands	Puff duration	Analysis of Youtube videos of nine conventional smokers and 64 e-cigarette users	Longer puff durations may help e-cigarette users to compensate for poor nicotine delivery
		Exhalation duration	
Vansickel and Eissenberg (75)	Own brand	Plasma nicotine	Blood samples were collected at baseline (0 h), after 10 puffs, after 1 h *ad libitum* puffing, and after a 2-h rest period	Vaping and smoking induce similar increases in plasma nicotine and heart rate
		Heart rate	
		Experienced e-cigarette users (*n* = 8)	
**STUDIES REPORTING NEGATIVE IMPACT OF E-CIGARETTES AND VAPING AS COMPARED TO CONVENTIONAL CIGARETTES AND SMOKING**				
Westenberger (4)	Njoy Smoking everywhere	Nicotine content in solutions and mist	HPLC-UV	The nicotine in several e-cigarette solutions is too variable to be considered safe. The amount of nicotine delivered per puff is inconsistent
*FDA Study*		
Hadwiger et al. (57)	Brand not indicated	Nicotine content in solutions	HPLC-UV	Presence of nicotine in products labeled as containing no nicotine
*FDA Study*	
Trtchounian et al. (84)	Liberty stix Smoking everywhere Njoy Crown seven versus eight brands of conventional cigarettes	Vacuum required for each puff Aerosol or smoke density	Manometer coupled to smoking machine Absorbance measurement using a spectrophotometer	More vacuum required to vape than to smoke. Smoke and aerosol density remained stable for conventional and e-cigarettes over the first 10 puffs. Aerosol density for e-cigarettes gradually decreased as e-cigarette life extended to 300 puffs. This is reflected by a gradual increase in vacuum required for each puff on the e-cigarette.
Trehy et al. (58) *FDA Study*	Njoy	Nicotine content in solutions and nicotine delivery/puff	HPLC-UV Smoking machine	Nicotine in e-cigarette cartridges and refill solutions is inaccurately labeled and nicotine content varies by manufacturer. The amount of nicotine delivered per puff is consistent.
	Smoking everywhere	
	CIXI			
	Johnson Creek	
Trtchounian and Talbot (65)	Njoy Ncig Liberty stix Crown seven Smoking everywhere VapCigs	Design flaws and defective parts	E-cigarettes were purchased online. Information about the parameters inspected was obtained via e-mail to vendors or by visual inspection of the product and product literature	Design flaws, lack of adequate labeling, concerns about control and health issues argue for removal of e-cigarettes from the market
		Labels on cartridges, and wrappers	
		Leakiness of cartridges	
		Disposal documentation	
		Errors in filling of mail orders	
		Instruction manual Truth in advertisement	
Williams and Talbot (86)	Various brands	Airflow rate required to produce aerosol Pressure drop Aerosol density	Airflow meter Manometer Absorbance measurement using a spectrophotometer	Significant variability exists between and within brands of e-cigarettes in the airflow rate required to produce aerosol, the pressure drop, the length of time cartridges last, and production of aerosol
Cheah et al. (60)	Twenty different brands of e-cigarettes	Nicotine content in cartridges E-cigarettes quality and documentation	GC-MS Visual inspection of the product and product literature	Variable nicotine content in cartridges of the same brand, and inconsistency with product labeling, along with misleading information on labels raises concern about e-cigarette safety
Cameron et al. (62)	Brands of e-cigarette nicotine solutions and cartridges tested include …	Nicotine content in solutions and cartridges	LC-MS	Nicotine levels in e-cigarette solutions were too variable to be considered safe
	Vapor liquid	
	No Brand liquid	
	Smart Smoke liquid	
	BE112 cartridge	
	Vapor cartridge	

HPLC-EC, high performance liquid chromatography – electrochemical detection; GS, gas chromatography; LC, liquid chromatography; GC-TSD, gas chromatography – thermionic specific detection; HPLC-UV, high performance liquid chromatography – ultraviolet detection; GS-MS, gas chromatography – mass spectrometry; LC-MS, liquid chromatography-mass spectrometry; NRT, nicotine replacement therapy.

## Clinical and Physiological Effects of Acute Vaping

The harmful effects of smoking on human health are obvious and well documented. In contrast, effects of vaping on human health are inconclusive due to the extreme paucity of empirical research investigating the presence of vaping-induced health hazards and/or benefits. Few studies have actually reported deleterious effects of vaping. In one report, McCauley et al. (99) present a case study concerning a 42-year-old woman diagnosed with exogenous lipoid pneumonia due to e-cigarette use. She presented with a 7-month history of dyspnea, productive cough, and fevers which coincided with her use of e-cigarettes. Samples of her sputum, and bronchoalveolar lavage revealed lipid-laden macrophages. Glycerin, an ingredient added to e-cigarette solutions for the purpose of producing visual smoke when vaping, was thought to be the causative agent. Computed axial tomography (CAT) images of her lungs revealed areas of patchy ground glass superimposed on interlobular septal thickening, a pattern typical of a restrictive ventilatory defect with diffusion impairment, and consistent with the patient’s diagnosis. Cessation of e-cigarette use resulted in improvement of her symptoms that was verified by follow-up lung radiography, however, pulmonary function testing still indicated mild diffusion impairment. Since the case study does not reveal if the patient is a current or ex-smoker and for how long, it is unclear whether the persistent diffusion impairment is a result of a concurrent or previous smoking habit, the use of e-cigarettes, or the after effects of lipoid pneumonia *per se*. In another report, Vardavas et al. (100) found that 5 min of acute vaping among healthy smokers had no effect on basic pulmonary parameters [i.e., forced expiratory volume in 1 s (FEV_1_), forced vital capacity (FVC), peak expiratory flow (PEF), or midexpiratory flows at 50 (MEF_50_) and 75 (MEF_75_) percent]. This is in agreement Flouris et al. (79) who reported the FEV_1_/FVC ratio after acute vaping to be non-significantly reduced by 3.0% (79). This study, also reported the FEV_1_/FVC ratio after acute tobacco smoking to be significantly reduced by 7.2%. Vardavas et al. (100) did find decreased amounts of exhaled nitric oxide and increased peripheral airway resistance and impedance in smokers who vaped for 5 min. From these results they concluded that acute vaping has “immediate adverse physiological effects similar to some of the effects observed with smoking” but that the long-term health effects of vaping are not known and potentially harmful. The authors went on to qualify their conclusion by stating that although the differences in exhaled nitric oxide, airway resistance, and impedance were statistically significant, the differences are probably not clinically important. It is possible that the increased airway resistance and impedance demonstrated by Vardavas et al. (100) is partially due to the nicotine inhaled from the e-cigarettes. Evidence for this is seen in a study reporting that non-smokers who inhaled nicotine (0–64 mg/ml) showed a dose-dependent increase in both the amount of coughing and airway obstruction, suggesting that nicotine stimulates afferent nerve endings in the bronchial mucosa which then triggers parasympathetic cholinergic pathways leading to bronchoconstriction (101).

In recent years there has been an effort to clinically use exhaled nitric oxide as an important non-invasive adjunct to pulmonary function testing (102) in order to monitor the degree of airway inflammation and eosinophilia (103, 104) commonly observed in conditions such as asthma. Unfortunately, interpretation of exhaled nitric oxide levels in the clinical setting is complex and confusing requiring adjustments for gender, age, height, respiratory infection, allergies, and smoking (105, 106). Given these difficulties, its validity is controversial. The major consensus in the literature is that the amount of exhaled nitric oxide is reduced in long-time smokers, as compared to non-smokers (105–109). In addition, it has been shown that smoking cessation is associated with an increase in exhaled nitric oxide back toward non-smoker levels (110). A possible mechanism of action for the opposing relationship of exhaled nitric oxide in smokers versus non-smokers could be the high levels of carbon monoxide present in cigarette smoke since there is strong evidence suggesting that carbon monoxide inhibits nitric oxide production by blocking nitric oxide synthase activity (111, 112). This mechanism is unlikely to occur with long-term vaping since carbon monoxide levels in e-cigarette mist are negligible (9). In any case, there is no available literature showing the long-term effects of vaping on exhaled nitric oxide or carbon monoxide, although Caponnetto et al. (113) did show exhaled carbon monoxide levels to decrease from 31 to 4 ppm, 29 to 2 ppm, and 35 to 5 ppm in three individuals who first successfully transitioned from conventional cigarettes to e-cigarettes and then quit e-cigarettes altogether. The first time carbon monoxide was measured, all individuals were heavy smokers (45 pack/year, 28 pack/year, and 89 pack/year histories). The final time that exhaled carbon monoxide was measured all individuals had been smoke- and vape-free for nearly 2 years. Using an experimental group of “healthy” smokers, Vardavas et al. (100) reported a decreased fraction of exhaled nitric oxide (FENO) after just 5 min of vaping (from 13.02 to 10.89 ppb) which they correlated with airway inflammation and oxidative stress. In their introduction they state that “smokers have significantly lower concentrations of FENO – a non-invasive marker of bronchial inflammation – compared with non-smokers.” On the other hand, in their discussion they state that “nitric oxide is an additional marker that has been implicated in the pathophysiology of airway diseases associated with smoking, is strongly correlated with eosinophilic inflammation and bronchial hyperactivity, and has become an established marker for assessing oxidative stress, indicating the immediate effect e-cigarette usage might have on pulmonary homeostasis.” From these two statements and from their FENO results, it is unclear whether they mean that smoking and vaping produce less bronchial inflammation and oxidative stress or more bronchial inflammation and oxidative stress when compared to not smoking or not vaping.

Although base levels of nitric oxide tend to be lower in smokers compared to non-smokers, Chambers et al. (114) observed significant increases in exhaled nitric oxide from 2.6 ppb before smoking to 4.8 ppb 1 min and 3.2 ppb 10 min after smoking a cigarette. Buda et al. (115) reported FENO to be 18, 29, and 16% higher than baseline 30, 45, and 60 min after smoking a cigarette, respectively. The findings of Vardavas et al. (100) concerning FENO levels after acute vaping are in direct opposition to what has been observed immediately after smoking a cigarette (114, 115). These results clearly demonstrate that acute vaping and acute smoking affect pulmonary nitric oxide metabolism, and the associated airway inflammatory responses, differently. From the available literature it is not clear how vaping might affect pulmonary inflammatory processes, but, as previously indicated, glycerin has been linked to lipoid pneumonia (99), and nicotine is known to generate endothelial dysfunction and systemic inflammation (16). Propylene glycol mist has been shown to produce ocular and respiratory irritation (116), and increase the risk of acquiring asthma (117), although Robertson et al. (118) reported that long-term inhalation of propylene glycol vapor by both monkeys and rats produced no deleterious pulmonary effects and Laugesen et al. (9) found no ill effects in humans. Bahl et al. (119) investigated the effects of a number of e-cigarette refill fluids on cultured human embryonic stem cells (hESCs), and human pulmonary fibroblasts (hPFs) and found that nicotine in e-cigarette refill fluids had no effect on hESC or hPF cytotoxicity at any concentration. However, they did report a positive correlation between hESC cytotoxicity, and the number and concentration of other chemicals used to flavor e-cigarette refill fluids. Similar results were published by Romagna et al. (120) who demonstrated that an extract of e-cigarette mist was less cytotoxic to cultured murine fibroblasts than an extract of tobacco cigarette smoke. A further indication that there are differences in the inflammatory responses between vapers and smokers is illustrated in a study reporting an absence of increased inflammatory indices in smokers asked to vape for 30 min compared to smokers who were asked to smoke for 30 min (121). Acute smoking has long been known to increase white blood cell count, which is a sign of acute inflammatory load (122). Flouris et al. (121) were able to confirm elevations of white blood cell count, lymphocyte count, and granulocyte count in active smokers but not in active vapers. Support for this is seen in a recently published case report (123) where a 36-year-old male with a nine pack-year history of smoking exhibited reversal of chronic idiopathic neutrophilia symptoms after he quit smoking and started vaping. Table 4 summarizes the studies involving clinical and physiological effects of acute vaping.

**Table 4:** **Studies involving clinical and physiological effects of acute vaping**.

Authors (Reference)	Study design	Subjects	Study location	Key finding
**STUDIES REPORTING POSITIVE OR NEUTRAL IMPACT OF E-CIGARETTES AND VAPING ON HEALTH**				
Caponnetto et al. (113)	Three case reports	A 47-year old male A 38-year old female A 65-year old male	University of Catania, Catania, Italy	Participants successfully switched from conventional cigarettes to e-cigarettes and then quit e-cigarettes. Smoking cessation confirmed by exhaled carbon monoxide
Bahl et al. (119)	*In vitro* cultures	Human embryonic stem cells, and pulmonary fibroblasts	University of California, Riverside, California	Nicotine in e-cigarette refill fluids had no effect on the cytotoxicity of human embryonic stem cells
Flouris et al. (121)	Repeated-measures controlled study	Thirty human smokers (8 male) cycled through a control session, active smoking session, and active vaping session Fifteen never smokers (8 male) cycled through a control session, passive smoking session and passive vaping session	FAME Laboratory, Institute of Human Performance and Rehabilitation, Center for Research and Technology, Trikala, Greece	Acute smoking, but not acute vaping, induced increases in white blood cell count, lymphocyte count and granulocyte count
			University of Thessaly, Trikala, and Larissa, Greece	
			Palamas Health Center, Kardista, Greece	
			University of Botswana, Botswana	
			University of Crete, Crete, Greece	
			University of Wolverhampton, United Kingdom	
Farsalinos and Romagna (123)	Case report	A 28-year old male with chronic iodiopathic neutrophilia	Onasis Cardiac Surgery Center, Kallithea, Greece and Abich ABICH S.r.l. Toxicological Laboratory, Verbania, Italy	Smoking cessation and e-cigarette use reversed symptoms of chronic iodiopathic neutrophilia. Smoking cessation was confirmed by exhaled carbon monoxide
Flouris et al. (79)	Repeated-measures controlled study	Thirty human smokers (8 male) cycled through a control session, active smoking session and active vaping session. Fifteen never smokers (8 male) cycled through a control session, passive smoking session, and passive vaping session	FAME Laboratory, Institute of Human Performance and Rehabilitation, Center for Research and Technology, Trikala, Greece	Vaping produced smaller changes in pulmonary function but similar nicotinergic impact compared to smoking
Romagna et al. (120)	*In vitro* cultures	Murine Fibroblasts	ABICH S.R.L. Toxicological Laboratory, Verbania, Italy	Extract of e-cigarette mist is less cytotoxic than extract of cigarette smoke to murine fibroblasts
**STUDIES REPORTING NEGATIVE IMPACT OF E-CIGARETTES AND VAPING ON HEALTH**				
Bahl et al. (119)	*In vitro* cultures	Human embryonic stem cells, and pulmonary fibroblasts	University of California, Riverside, California	The number and concentration of chemicals (other than nicotine) used to flavor e-cigarette refill fluids increased cytotoxicity
McCauly et al. (99)	Case report	A 42-year woman with exogenous lipoid pneumonia	Legacy Good Samaritan Medical Center, Portland, Oregon	Termination of e-cigarette use cleared the exogenous lipoid pneumonia
Vardavas et al. (100)	Laboratory-based intervention study	Active vaping (experimental) for 5 min in smokers (14 men) versus passive vaping (control) for 5 min in 10 smokers randomly selected from the experimental group	Participants from a community in Athens, Greece	Five minutes acute vaping-induced a decrease in exhaled nitric oxide, and an increase in airway resistance and impedance in experienced smokers

## Conclusion

Despite the popularity e-cigarettes have gained worldwide, very little rigorous research has been done regarding the effects these devices have on human health. This article reviews the existing evidence-based literature, dealing with surveys soliciting personal views on vaping; studies analyzing potential toxins and contaminants in e-cigarette cartridges, solutions, and mist; reports profiling nicotine content, delivery, and pharmacokinetics; and clinical and physiological studies investigating the effects of acute vaping. When compared to the harmful effects of smoking, these studies suggest that vaping could be used as a possible “harm reduction” tool. There is evidence supporting e-cigarettes as an aide for smoking cessation, at least as successful as currently available FDA-approved NRTs. Less evidence exists to suggest that e-cigarettes are effective in recovery from nicotine dependence. More rigorous research is essential before any solid conclusions can be drawn about the dangers, or usefulness of e-cigarettes. In particular, more rigorous research is required delving into both acute and long-term cardiopulmonary effects of vaping, especially those experiments comparing the effects of vaping with those of smoking. E-cigarettes are fast becoming a new “tobacco” industry (124) that could reduce the incidence of traditional smoking. It is also possible that e-cigarettes may either decrease or increase the incidence of nicotine addiction. Given these uncertainties, will the availability of e-cigarettes provide for healthier U.S. and world populations, as harm reductionists hope, or will other more dangerous ill effects ultimately emerge? Health care professionals must remain current with the literature concerning e-cigarettes and vaping. Only then can they make informed decisions aimed at maximizing human safety and minimizing the potential ill effects e-cigarettes may have on their patients and the public. Only then can the new challenge regarding e-cigarettes and vaping in clinical medicine and public health be adequately addressed.

## Conflict of Interest Statement

The author declares that the research was conducted in the absence of any commercial or financial relationships that could be construed as a potential conflict of interest.
